# Effect of Various Compounds Blocking the Colony Pigmentation on the Aflatoxin B1 Production by *Aspergillus flavus*

**DOI:** 10.3390/toxins8110313

**Published:** 2016-10-28

**Authors:** Vitaly G. Dzhavakhiya, Tatiana M. Voinova, Sofya B. Popletaeva, Natalia V. Statsyuk, Lyudmila A. Limantseva, Larisa A. Shcherbakova

**Affiliations:** All-Russian Research Institute of Phytopathology, Bolshie Vyazemy, Moscow 143050, Russia; dzhavakhiya@yahoo.com (V.G.D.); tatiana.voinova@bk.ru (T.M.V.); unavil@yandex.ru (S.B.P.); lutik47@yandex.ru (L.A.L.); larisavniif@yahoo.com (L.A.S.)

**Keywords:** aflatoxin B1, fungal melanins, polyketide biosynthesis, compactin, thymol, fluconazole, 3-hydroxybenzaldehyde, *Aspergillus flavus*

## Abstract

Aflatoxins and melanins are the products of a polyketide biosynthesis. In this study, the search of potential inhibitors of the aflatoxin B1 (AFB1) biosynthesis was performed among compounds blocking the pigmentation in fungi. Four compounds—three natural (thymol, 3-hydroxybenzaldehyde, compactin) and one synthetic (fluconazole)—were examined for their ability to block the pigmentation and AFB1 production in *Aspergillus flavus*. All compounds inhibited the mycelium pigmentation of a fungus growing on solid medium. At the same time, thymol, fluconazole, and 3-hydroxybenzaldehyde stimulated AFB1 accumulation in culture broth of *A. flavus* under submerged fermentation, whereas the addition of 2.5 μg/mL of compactin resulted in a 50× reduction in AFB1 production. Moreover, compactin also suppressed the sporulation of *A. flavus* on solid medium. In vivo treatment of corn and wheat grain with compactin (50 μg/g of grain) reduced the level of AFB1 accumulation 14 and 15 times, respectively. Further prospects of the compactin study as potential AFB1 inhibitor are discussed.

## 1. Introduction

Fungal polyketides represent a large group of biologically active compounds synthesized by enzymes from the polyketide synthase (PKS) family [1]. Aflatoxins are secondary metabolites produced by *Aspergillus flavus* via the polyketide pathway. The ability to produce aflatoxins is probably not related to the essential features of *A. flavus*, but may give some competitive advantages to this fungus compared with other toxin-sensitive microorganisms [2,3]. A highly hepatotoxic and carcinogenic aflatoxin B1 (AFB1) is of greatest concern because it can contaminate a wide range of agricultural products that results in great losses in agricultural income and has a significant negative economic impact. A number of methods are developed for pre- and post-harvest aflatoxin management [4]. Traditional approaches, such as fungicides, have shown very limited success in the AFB1 control [5]. Therefore, the search and development of efficient preparations able to inhibit AFB1 production still remains of great theoretical and practical importance.

Fungal melanins, representing high-molecular hydrophobic pigments, are also synthesized via the PKS-depending pathway [6]. The blocking of the melanin biosynthesis in pathogenic fungi can result in the loss of their pathogenicity and an increased susceptibility of fungi to biotic and abiotic stresses [7,8,9]. For example, a defect in one of the PKS-encoding genes of *A. flavus* resulting in a formation of non-pigmented sclerotia significantly increased the susceptibility of the fungus to the deleterious effect of UV light and heat; in addition, the melanin-deficient mutant was less resistant to insect predation as compared with the wild-type strain generating melanized sclerotia [10].

Based on the data of experiments with radiolabeled polyketide intermediates, the following simplified scheme of AFB1 biosynthesis was proposed: acetate → hypothetic polyketide intermediates → norsolorinic acid → averantin → averufin → versiconal hemiacetal acetate → versicolorin A → sterigmatocystin → AFB1 [11]. Later, this scheme was significantly enlarged. To date, AFB1 biosynthesis is considered to include 23 enzymatic reactions, and 15 intermediates of this pathway have been identified [12].

Norsolorinic acid is the first stable intermediate in the aflatoxin biosynthesis. A structural similarity between the pigments of *A. flavus* spores and norsolorinic acid made it possible to suppose that these pigments and aflatoxins have common precursors; therefore, aflatoxin and melanin biosynthetic pathways have common initial stages [13]. Due to this fact, the search for compounds able to block the early stages of the polyketide biosynthetic pathway before its branching to the aflatoxin and melanin biosyntheses represented a promising task, since such inhibitors would prevent AFB1 accumulation in treated food and feed products and simultaneously decrease the contamination of these products with *A. flavus* due to the decreased viability of melanin-deficient fungus.

In our earlier studies, we tested various phosphoanalogues of amino acids and their derivatives for their ability to block different stages of the polyketide biosynthetic pathway. As a result, some compounds, which blocked either AFB1 or melanin biosynthesis, have been revealed; in the last case, a simultaneous stimulation of the toxinogenesis was observed [14,15]. However, none of these compounds was able to block early stages of polyketide biosynthesis, i.e., simultaneously inhibit the production of AFB1 and melanin.

This study continued the search for inhibitors of the early stages of toxinogenesis among natural and synthetic compounds, which, as we found earlier, are able to block the pigmentation of some plant pathogenic fungi. Our earlier study showed that compactin, a natural inhibitor of the sterol biosynthesis, which inhibits HMG-CoA reductase catalyzing the conversion of HMG-CoA into mevalonic acid [16], causes the depigmentation of colonies of several plant pathogenic fungi [17]. Another possible inhibitor of AFB1 biosynthesis could be fluconazole, a synthetic triazole-based fungicide. It is known that the fungicide action of triazoles is determined by their ability to inhibit the biosynthesis of sterols [18]. In the preliminarily study, we showed that this fungicide was able to depigment the colonies of some fungi including *A. flavus*, so we supposed it could also influence on toxinogenesis.

According to one of the existing hypotheses, the evolution of plants, which are infected with toxigenic fungi, could result in their ability to produce specific compounds inhibiting the biosynthesis of toxins [19]. In fact, some plant terpenoids are really able to suppress AFB1 production by *A. flavus* [20]. One of such compounds is thymol, widely used in medicine, veterinary, and plant protection, so we included this compound into this study.

It is known that 2-chloroethyl phosphoric acid inhibits AFB1 biosynthesis due to oxidative stress alleviation [21]. Some other studies showed an important role of oxidative stress at the initial stages of AFB1 biosynthesis, though the mechanisms of this effect and the specific forms of reactive oxygen influencing on the toxinogenesis still remain unclear [22]. The earlier study devoted to the chemosensitization of *Aspergillus* spp. and some other fungi to the action of various antifungal agents showed that sensitizers, which can act as antioxidants preventing the oxidative stress, increased the sensitivity of fungi to industrial fungicides [23]. Among the tested compounds, the most active chemosensitizer was 3-hydroxybenzaldehyde (3-HBA), a compound of a plant origin. Since the antioxidant properties of this compound could probably inhibit the toxigenesis in *A. flavus*, it was also included into the current study.

**Figure 1:**
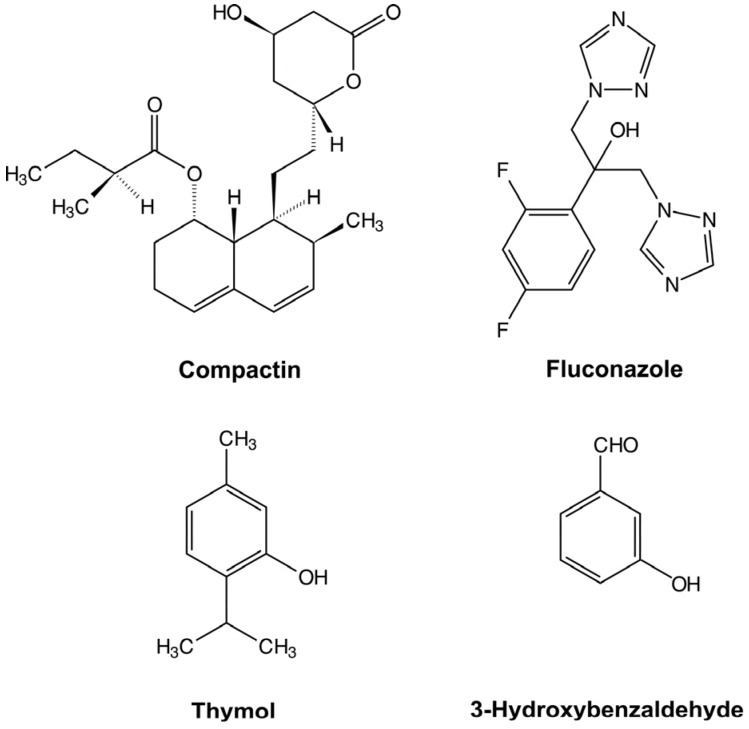
Compounds used in the study.

Thus, based on the above-described considerations, the purpose of this study was the examination of four compounds of different nature (Figure 1) for their effect on both AFB1 and melanin production to find compound(s) able to block the early stages of polyketide biosynthesis, as well as the in vivo evaluation of the AFB1 accumulation in wheat and corn grain infected with toxigenic *A. flavus* and treated with the most efficient compound.

## 2. Results

### 2.1. Effect of Tested Compounds on the Pigment Production in Aspergillus flavus

**Figure 2:**
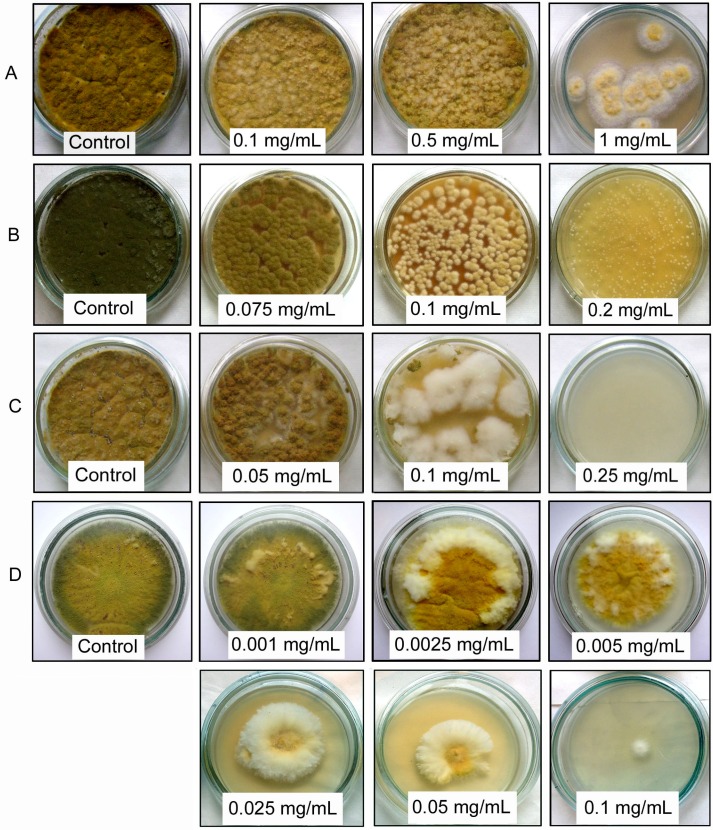
Effect of various compounds on the colony growth and pigmentation of *Aspergillus flavus*. (**A**) 3-hydroxybenzaldehyde; (**B**) thymol; (**C**) fluconazole; (**D**) compactin.

According to the obtained results, all tested compounds were able to suppress the pigment production in *A. flavus* (Figure 2).

The maximum effect was observed for compactin; the mycelium changed its color from green to yellow starting from the compactin concentration equal to 2.5 μg/mL, while no significant growth suppression was revealed (Figure 2, Table 1). Such a picture was observed for either stab-inoculation into the center of a Petri dish or the spreading of spore suspension on the whole agar surface. Taking into account changes in the colony diameter in the case of the stab-inoculation, the compactin concentration providing the fungal growth suppression (25 μg/mL) is ten times greater than the concentration, at which mycelium begins to change its color (2.5 μg/mL).

In the case of a stub-inoculation of control (inhibitor-free) Petri dishes, *A. flavus* grows as coalesced small pigmented colonies, which is caused by abundant sporulation accompanied by a discharge of spores onto nutrient medium with their further germination. In the case of compactin-containing medium, the situation is quite different starting from the compactin concentration equal to 25 μg/mL. After a stub-inoculation, only one discolored colony grows in the center of a Petri dish (Figure 2). This phenomenon was observed for all compactin concentrations causing a full discoloration of mycelium (>25 μg/mL) and is explained by the inhibition of the spore generation on aerial mycelium, since the morphological examination of the mycelium under a light microscope did not reveal any spores in colonies grown on compactin-containing medium. In the case of the compactin concentration range 1–10 µg/mL, characterized by incomplete mycelium discoloration, the microscoping of colonies showed the presence of conidia and spores, but their color was clarified as compared with the control.

**Figure 3:**
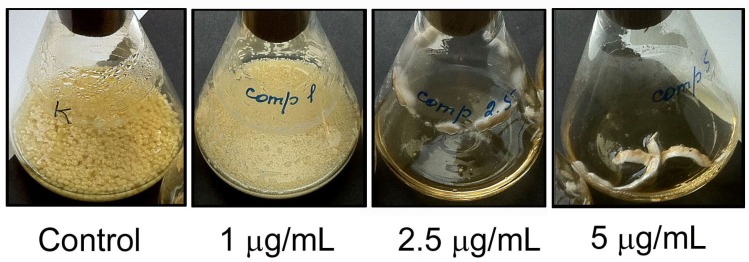
Effect of various compactin concentrations on the mycelium growth of *Aspergillus flavus* on liquid medium.

In the case of the *A. flavus* cultivation on the liquid compactin-containing medium, it was difficult to evaluate the effect of compactin on the mycelium pigmentation, since the fungal growth was significantly suppressed, when the compactin concentration exceeded 1 μg/mL. The dry weight of mycelium in the control variant and at the compactin concentrations of 1, 2.5, and 5 μg/mL was 1.53 ± 0.04, 1.33 ± 0.34, 0.10 ± 0.04, and 0.12 ± 0.03 g, respectively; a weak discoloration was observed at the compactin concentration of 1 μg/mL (Figure 3).

### 2.2. Effect of Tested Compounds on the Aflatoxin Production by Aspergillus flavus

**Figure 4:**
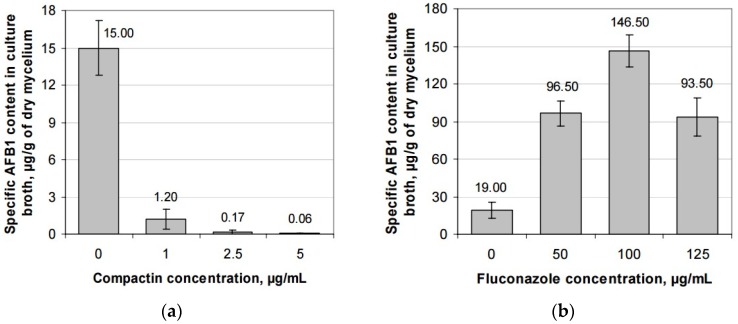

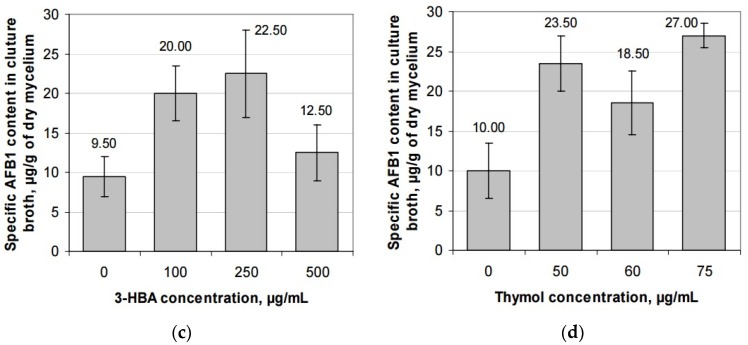
Effect of the tested compounds on the aflatoxin B1 production by *Aspergillus flavus*: (**a**) compactin; (**b**) fluconazole; (**c**) 3-hydroxybenzaldehyde; (**d**) thymol.

The effect of the tested compounds on AFB1 production by *A. flavus* grown in liquid medium is shown in Figure 4. According to the obtained data, fluconazole fungicide added to the liquid medium at the concentrations of 50–100 μg/mL several times increased AFB1 production by *A. flavus*. An increased AFB1 production was also observed in the case of thymol and 3-HBA. Thus, the effect of these three compounds was similar to that observed earlier for *N*-hydroxyputrescine and (1-aminoethyl)phosphonic acid [24].

Unlike other compounds, compactin significantly reduced the AFB1 accumulation in culture broth. The calculated coefficient of the correlation between the compactin concentration and specific AFB1 content in culture broth was –0.646, which, according to the Chaddock scale, indicates a significant negative correlation between the mentioned parameters. Therefore, a conclusion can be made that compactin inhibits AFB1 production in *A. flavus* more actively than it suppresses the growth of mycelium. The experimental data confirm this conclusion: for example, the presence of 1 μg/mL of compactin almost did not suppress the fungal growth, while significantly reducing the toxin production (Figure 3 and Figure 4).

### 2.3. In Vivo Evaluation of the Aflatoxin B1 Content in Treated Grain Samples

**Figure 5:**
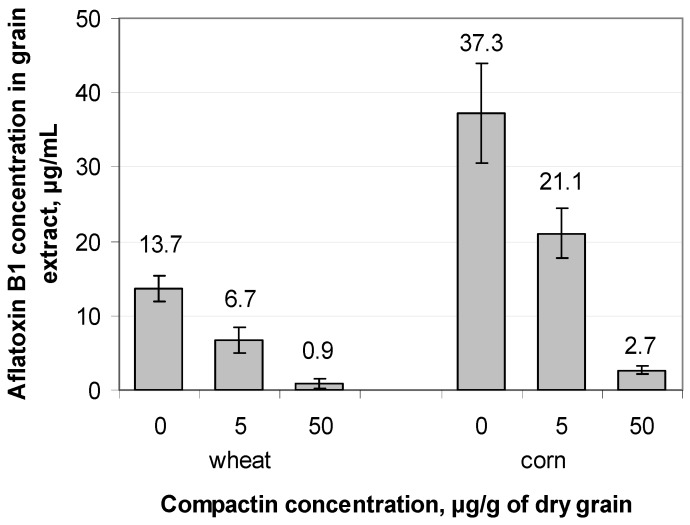
Aflatoxin B1 content in extracts from wheat and corn grain treated with compactin and artificially infected with toxigenic *Aspergillus flavus*.

The data on the effect of the preliminary treatment of corn and wheat grain with compactin on the AFB1 accumulation in grain infected with *A. flavus* are shown in Figure 5.

In both cases, AFB1 concentration in extracts obtained from compactin-treated grain was significantly lower than that in the control reducing up to 6%–7% for the maximum applied compactin concentration (50 μg/g of dry grain). The calculated correlation coefficients between the applied compactin concentration and the extracted AFB1 were –0.93 and –0.90 for corn and wheat, respectively.

**Figure 6:**
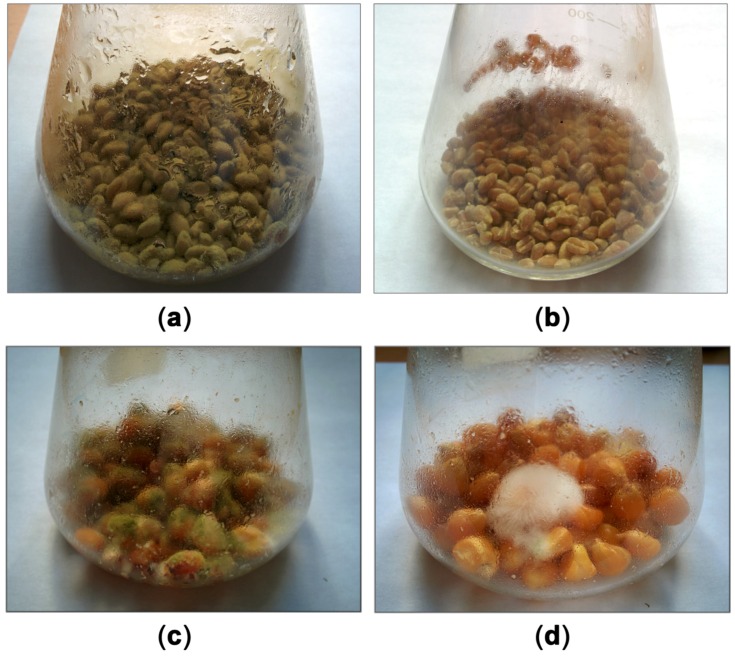
Development of *Aspergillus flavus* on grain treated with compactin (5 μg/g of dry grain): (**a**) control and (**b**) treated wheat grain; (**c**) control and (**d**) treated corn grain.

A difference between the growth of the fungus on treated and untreated grain was clearly visible (Figure 6), especially in the case of corn. The surface of untreated grains was covered by green colonies, whereas, in the case of the treated grain, less developed white mycelium was observed, which did not contain mature pigmented conidia.

## 3. Discussion

To date, there have been many studies describing various natural and synthetic compounds that have been shown to be specific inhibitors of AFB1 production. The majority of these compounds are of plant origin [20,25]. There are also some microbial metabolites, such as the melanin pigment from *Streptomyces torulosis* [26], some other metabolites from *Streptomyces* sp. [27], and respiration inhibitors of a fungal origin, characterized by the ability to significantly reduce AFB1 production by *A. flavus* without any significant effect on the fungal colony growth [28]. It is also known that some synthetic inhibitors of the pentaketide biosynthesis of melanin, such as the tricyclazole fungicide, are also able to inhibit AFB1 biosynthesis presumably via the inhibition of reductase involved in the versicolorin A conversion to dimethyl-sterigmatostycin at the late stages of AFB1 biosynthesis [29].

In this and previous studies, we examined a hypothesis about the possibility to reveal effective inhibitors of the early stages of AFB1 biosynthesis among compounds able to block the pigmentation of the fungus. As far as the authors know, compounds able to simultaneously inhibit both melanin and AFB1 production have only been described in a few publications [29,30], but no any special study of melanogenesis inhibitors was carried out.

Three of four compounds tested in this study suppressed the biosynthesis of the fungal pigment and, at the same time, stimulated the toxinogenesis. We also revealed several such compounds in our previous studies [14,24]. This effect can be explained by the common initial biosynthetic stages of both metabolites first hypothesized by Brown and Salvo [13]. At the later stages, the polyketide biosynthetic chain may branch in different pathways resulting in the production of melanin and AFB1. In this case, the observed stimulation of AFB1 production may be explained in the following way: these compounds block the melanin production at the stages located after the branching point that promotes the accumulation of preceding intermediates, which are also AFB1 precursors. An increased precursor accumulation provides increased AFB1 production.

The last tested compound, compactin, was able to suppress both pigment and toxin formation. One of the possible explanations for this is that it blocks the polyketide biosynthetic chain prior to the branching point; another possibility is that compactin is able to simultaneously block both melanin and AFB1 biosynthetic chains after the branching point. A more detailed study is required to determine if any of these variants is correct.

All inhibitors of the colony pigmentation tested in our current and previous studies showed either a stimulating or an inhibiting effect on AFB1 biosynthesis. Therefore, our data probably confirms the interrelation between the aflatoxinogenesis and the melanin biosynthesis in *A. flavus* occurring via the DOPA pathway [6] and can be used for the further elucidation of the early stages of toxinogenesis. The revealed interrelation can probably be used for practical purposes: the examination of potential AFB1 inhibitors for their ability to block melanin production can serve as a preliminary screening stage able to reveal highly effective toxin inhibitors, such as compactin. In addition, efficient AFB1 inhibitors could be probably revealed among the known inhibitors of the melanin biosynthesis.

The analysis of existing scientific and patent publications showed a lack of any information about the ability of compactin or other statins to inhibit the biosynthesis of mycotoxins. Therefore, we probably revealed a new group of AFB1 inhibitors that opens wide prospects for future studies. Among the planned theoretical tasks, we can mention the determination of the exact point of compactin action in the polyketide biosynthetic pathway using known intermediates and the study of the compactin effect on other mycotoxins of the polyketide origin, such as zearalenone. The authors also plan to continue the study of other statins for their potential as AFB1 inhibitors and have already obtained promising results for lovastatin [24]. Note that, according to the obtained data, compactin is able to inhibit the toxinogenesis at very low concentrations. Finally, an in vivo study of the compactin ability to efficiently reduce AFB1 accumulation in wheat and corn grain contaminated with toxigenic *A. flavus* demonstrated a potential for the practical application of this compound. Since the treatment of feed using such concentrations of active substances does not require high-purity compactin, the technology of its isolation and purification from culture broth can be simplified to reduce the cost of the final product. According to our calculations, the cost of such partially purified compactin obtained using our overproducing strain should be about 50 USD/kg. Taking into account the working compactin dose of 0.1 mg/kg of grain, the final cost of the grain treatment with compactin will make about 5 USD/ton.

In our further work, we plan to study some practical aspects of the possible statin application as AFB1 inhibitors for feed treatment, such as the evaluation of the duration of their inhibiting effect and toxicity assay towards birds and mammals.

## 4. Materials and Methods

### 4.1. Strains, Chemicals, and Culture Conditions

*Aspergillus flavus* AF11 (VKM F-27) was used as a producer of AFB1 throughout the study. The strain was purchased in the All-Russian Collection of Microorganisms (Skryabin Institute of Biochemistry and Physiology of Microorganisms, Pushchino, Russia). AF11 was maintained on agar medium of the following composition (g/L): agar, 20; yeast extract, 5; glucose, 20. In addition, a microelement stock solution (FeSO_4_·7H_2_O (24 mg/mL), MnCl_2_·4H_2_O (42 mg/mL), CuCl_2_·2H_2_O (12 mg/mL), ZnSO_4_·7H_2_O (84 mg/mL)) was added at a ratio of 100 μL per 1 L of the agar medium. The same medium was used to observe mycelium color changes.

A spore suspension prepared from a 10-day-old culture at a concentration of 1 × 10^7^ spores/mL was used as the inoculum. Spore suspension (1 mL) was inoculated into 250 mL shaken flasks containing 50 mL of liquid Payne–Hagler medium [31]. Before the inoculation, the medium was supplemented with solutions of tested compounds sterilized by a filtration through 0.22 μm Millipore membrane filters (MilliporeSigma, St. Louis, MO, USA). In the case of control flasks, the medium did not contain potential inhibitors. The culture was incubated at 27 °C for 170 h under aeration conditions on a New Brunswick™ Excella E25/25R incubation shaker (New Brunswick Scientific, Edison, NJ, USA) at 200 rpm and a 5 cm orbit.

Thymol, fluconazole, and 3-HBA were purchased from Medsi LLC (Moscow, Russia), Makiz-Pharma LLC (Moscow, Russia), and Sigma-Aldrich (St. Louis, MO, USA), respectively.

Compactin was obtained using an overproducing *Penicillum citrinum* 18–12 strain developed by a multi-step induced mutagenesis. The cultivation of this strain and the compactin isolation and purification were carried out as described earlier [32]. Since compactin has poor water solubility, for our purposes, it was converted from the lactone form to the more soluble salt form. To do this, the known amount of compactin was dissolved in ethanol (the maximum concentration did not exceed 100 mg/mL), heated to a boil, then mixed with the equal volume of the equimolar NaOH solution and cooled to the room temperature. Then, the solution was diluted to the required concentration; pH of the final solution should be about 7.0.

The concentration range of the tested substances included minimal working concentrations providing a significant effect on the melanin production.

### 4.2. Analysis of Aflatoxin B1

After the completion of cultivation, culture broth of each experimental or control flask was centrifuged at 8000 *g* to separate the mycelia. The mycelia were washed with 3 mL of distilled water twice and dried at room temperature up to a constant weight.

The AFB1 content in the supernatant of culture broth was determined by HPLC as described earlier [33]. Aflatoxin B1 purchased from Sigma-Aldrich (St. Louis, MO, USA) was used as a reference sample.

To exclude the possible effect of fungal growth inhibition by the tested compounds, their inhibiting effect was assessed by the specific AFB1 content in culture broth (AFB1 content in culture broth normalized to the dry weight of mycelium). The effect of tested compounds on AFB1 biosynthesis was determined by a comparison between experimental and control flasks.

### 4.3. Evaluation of the Aflatoxin B1 Content in Treated Grain Samples

To assess the ability of the most efficient tested compound to prevent in vivo AFB1 accumulation in grain, wheat (var. Zlata) and corn (var. Ross 140 SB) grain samples were used as the model objects. Twenty grams of wheat or corn grains were placed into 250 mL shaken flasks containing 10 or 20 mL of distilled water, respectively. Flasks were autoclaved for 1 h under a pressure of 0.5 atmosphere. Then, 1 mL of a sterile compactin solution (0.1 mg/mL or 1 mg/mL) or sterile distilled water (control) was added into the flasks. After the mixing of the flask content, each flask was inoculated by 1 mL of the spore suspension of *A. flavus* containing 1 × 10^7^ spores/mL. After the 8-day incubation at 26 °C, 50 mL of chloroform was added to each flask. Flasks were incubated for 3 h at 26 °C on a New Brunswick™ Excella E25/25R incubation shaker at 250 rpm, their content was then centrifuged for 30 min at 8000 *g* at room temperature to separate grain from the organic phase, and 200 μL of the chloroform extract was sampled from each flask. After the chloroform evaporation, the residue was dissolved in 200 μL of methanol. The AFB1 concentration was further determined by HPLC as described above.

### 4.4. Data Analysis

Measurements were analyzed using a STATISTICA v. 6.1 software (StatSoft Inc., Tulsa, OK, USA) and depicted as their mean (over at least three independent experiments) ± standard deviation. Differences were considered significant when *p* < 0.05.

## Figures and Tables

**Table 1:** Effect of various compactin content in agar medium on the growth of *Aspergillus flavus* colonies and the color of aerial mycelium.

Compactin Concentration, μg/mL	Colony Diameter, mm	Aerial Mycelium Color
0 (control)	90	Green
1	90	Green
2.5	90	Bright yellow/white
5	89	Bright yellow/white
10	88	Bright yellow/white
25	56	White with yellow center
50	45	White with yellow center
